# Zebrafish as a model for leukemia and other hematopoietic disorders

**DOI:** 10.1186/s13045-015-0126-4

**Published:** 2015-03-28

**Authors:** Parisa Rasighaemi, Faiza Basheer, Clifford Liongue, Alister C Ward

**Affiliations:** School of Medicine, Deakin University, Pigdons Road, Geelong, Victoria 3217 Australia; Centre for Molecular and Medical Research, Deakin University, Pigdons Road, Geelong, Victoria 3217 Australia

**Keywords:** Zebrafish, Leukemia, Myeloproliferative disorder, Myelodysplastic syndrome

## Abstract

Zebrafish is an established model for the study of vertebrate development, and is especially amenable for investigating hematopoiesis, where there is strong conservation of key lineages, genes, and developmental processes with humans. Over recent years, zebrafish has been increasingly utilized as a model for a range of human hematopoietic diseases, including malignancies. This review provides an overview of zebrafish hematopoiesis and describes its application as a model of leukemia and other hematopoietic disorders.

## Introduction

Zebrafish represents an alternative model for the study of vertebrate development and its perturbation, with powerful molecular, cellular, and genetic approaches available. This model has proven particularly useful for the study of hematopoiesis and its disruption, with zebrafish possessing all major blood cell types, which are generated by similar developmental pathways [1,2]. There is also strong conservation of transcription factors, signaling components, and functional proteins [3-7]. This review overviews zebrafish hematopoiesis and describes the application of this organism to the study of leukemia and other hematopoietic proliferative disorders.

## Review

### Zebrafish hematopoiesis

Like other vertebrates, zebrafish undergoes distinct waves of hematopoiesis [8]. At 10 h post fertilization (hpf), the ventral lateral mesoderm gives rise to hemangioblasts, bipotential cells expressing both hematopoietic (*scl*, *lmo2*, *gata2*), and vascular (*flk1*, *fli1*) transcription factors [9], which become further specified into both hematopoietic and endothelial cells [10].

**Figure 1:**
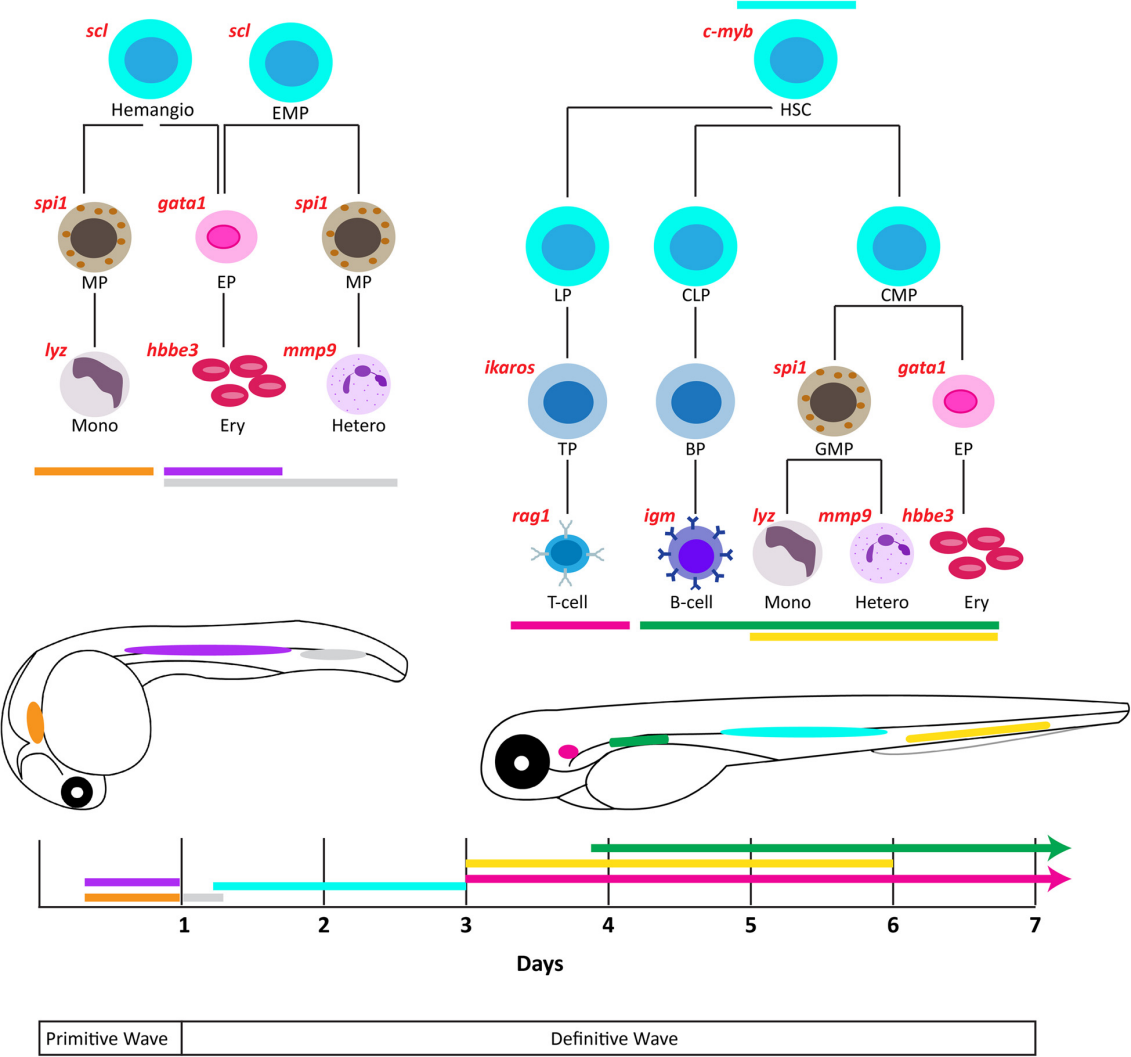
**Zebrafish hematopoiesis and its key regulators.** Schematic representation of hematopoiesis in zebrafish. The primitive wave commences in two locations, the anterior lateral mesoderm (ALM) (orange), which gives rise to primitive monocytes, and the intermediate cellular mass (ICM) (violet), which generates mostly primitive erythrocytes before 24 hpf. A transient ‘intermediate’ wave occurs in the posterior blood island (PBI) where both erythrocytes and heterophils are formed (grey). Definitive hematopoietic stem cells (HSCs) are initially formed by budding from the hemogenic endothelium on the ventral wall of dorsal aorta (blue). A subset of these HSCs migrate to the caudal hematopoietic tissue (CHT) (yellow) to produce several cell lineages, and also the thymus (purple), where T lymphocyte production occurs. Finally, HSCs seed the developing kidney (green), the final site of definitive hematopoiesis where erythroid, myeloid, and B lymphocyte production occurs. The lineage-specific transcription factors that serve to regulate this process are in red. Abbreviations: BP: B cell progenitor, CLP: common lymphoid progenitor, CMP: common myeloid progenitor, EP: erythroid progenitor, Ery: erythrocyte, GMP: granulocyte-monocyte progenitor, Hemangio: hemangioblast, Hetero: heterophil, HSC: hematopoietic stem cell, Mono: monocyte, TP: T cell progenitor.

Primitive hematopoiesis is initiated from these cells at two locations, the anterior lateral mesoderm (ALM) and the posterior lateral mesoderm (PLM) that later forms the intermediate cellular mass (ICM) (Figure 1) [11,12]. In the ALM, hemangioblasts differentiate into *spi1*^+^ myeloid precursors around 12 hpf, which migrate rostrally and then laterally across the yolk sac [13,14] and begin to express genes encoding cell surface colony-stimulating factor receptors *csf1r* [15] and *csf3r* [16]. These mature into distinct myeloid populations with differential expression of the genes for the myeloid-specific actin binding protein *l*-*plastin* [15] and the granulocytic enzyme myeloperoxidase (*mpo*) [17]. In the PLM, *gata1*^+^ erythroid precursors are generated at 12 hpf [18]. These commence expression of the erythropoietin receptor (*epor*) gene and differentiate into primitive erythrocytes expressing erythroid-specific genes including those encoding key enzymes (such as *alas2* and *carbonic anhydrase*) and globins (such as *hbbe3*) [19], which enter circulation around 24 hpf and persist until 4 dpf [1]. Around 24 hpf, a transient ‘intermediate’ wave of hematopoiesis occurs in the posterior blood island (PBI). Here, bipotent erythromyeloid progenitors (EMP) differentiate into *gata1*^+^ erythroid and *spi1*^+^ myeloid cells (Figure 1) [13,20].

Definitive hematopoietic stem cells (HSC) are initially formed at around 33 hpf by budding from the hemogenic endothelium on the ventral wall of the dorsal aorta, equivalent to the aorta-gonad-mesonephros (AGM) in mammals [21,22]. From 48 hpf, a subset of the *c*-*myb*^+^*runx1*^+^ HSCs expressing *cd44* migrate from the dorsal aorta to the caudal hematopoietic tissue (CHT), equivalent to the fetal liver in mammals [10]. Erythropoiesis commences at 3.5 dpf in the CHT as identified by expression of *gata1* and *hbbe3*, producing mature erythrocytes that gradually replace the primitive erythrocytes in circulation [23]. Definitive myelopoiesis similarly begins around 3 dpf in the CHT, as recognized by *l*-*plastin* expression [18].

By 54 hpf, some of the HSCs also migrate directly from the AGM to seed the thymus, the site of lymphopoiesis [24], developing into *ikaros*^+^ lymphoid progenitors [3]. By 4 dpf cells in the thymus express *rag1*, a transcription factor essential for lymphoid differentiation [25] and members of the interleukin-2 receptor (IL-2R) family (Sertori et al., submitted), with mature lymphocytes found in the thymus by 7 dpf [26]. Between 48 and 96 hpf, HSCs from the AGM and CHT also seed the marrow of the developing kidney, the ultimate site of definitive hematopoiesis, which is the equivalent of mammalian bone marrow [27,28]. These HSCs produce all hematopoietic lineages, including lymphoid progenitors that mature to B cells in the kidney from 28 dpf [29] and additional T cells in the thymus [1]. The thymus and kidney continue to generate hematopoietic cells throughout adulthood [10].

### Zebrafish as a model for leukemia and related disorders

In addition to the strong conservation of key developmental processes and genes, several other factors make zebrafish especially amenable to study the disruptions in hematopoiesis that underpin leukemia and related disorders. Genes can be easily ablated transiently using morpholino-mediated gene knockdown [30] or permanently using genome editing approaches, such as transcription activator-like effector nucleases (TALENs) [31] or clustered regularly interspaced short palindromic repeats (Cas9/CRISPR) [32], or targeting-induced local lesions in genome (TILLING) [33]. Alternatively, genes can be overexpressed transiently by introducing mRNA [34] or DNA [35], or stably through transgenesis, which extends to a variety of conditional/inducible approaches [36]. Sophisticated forward genetic screens are feasible in both zebrafish larvae and adult fish randomly mutated with chemical or transposon-mediated approaches to identify new genes involved in specific biological processes, including disease [37]. The transparent embryos, which develop externally and therefore are extremely accessible, provide exquisite opportunities for imaging [38]. These zebrafish embryo traits are also advantageous for large-scale drug screening by simple immersion as they are penetrable to small molecules and drugs [39,40]. These approaches have yielded new disease models that have increased our knowledge of the oncogenic process and uncovered novel therapeutics (Table 1).

**Table 1:** **Zebrafish models of leukemia and other hematological disorders**

**Disease model**	**Gene**	**Expression**	**Latency**	**Penetrance**	**References**
**AML**	Hs *RUNX1*-*ETO*	Ubiquitous	Embryonic	32–41%	[22]
		Ubiquitous - inducible (hs)	Embryonic (induced)	NS	[43,44]
	Hs *MOZ*/*TIF2*	Progenitor (*spi1*)	14–26 months	1%	[47]
	Hs *FLT3*	Ubiquitous	Embryonic	NS	[49]
	Hs *NPMc* ^+^	Ubiquitous	Embryonic	NS	[51]
	Mm *Mycn*	Ubiquitous - inducible (hs)	Embryonic (induced)	F0: 60% F2: 75%	[54]
	Dr *spi1* ^*G242D*^	Endogenous - hypomorph	3 days	NS	[57]
	Dr *ddx18*	Knockout	Embryonic	NS	[58]
**ALL**	Mm *Myc*	Lymphoid (*rag2*)	F0: 44–52 days F1: 32 days	F0: 5–6% F1: 100%	[2,34,59]
		Lymphoid (*rag2*) - inducible (cre)	151 days	13%	[61]
		Lymphoid (*rag2*) - inducible (cre/hs)	T-LBL: 120 days/T-ALL: 261 days	T-LBL: 81%/T-ALL: 100%	[62,63]
	Hs *MYC*	Ubiquitous - inducible (4HT)	4–5 weeks (induced)	100%	[66,68]
	Dr *pten*	Knockout	Embryonic	NS	[70,71]
	Hs *NOTCH1ic*	Lymphoid (*rag2*)	5 months	44%	[73]
	Hs *ETV6*-*RUNX1*	Ubiquitous	1 year	3%	[75]
	Dr *etv6*-*jak2a* (*ALL*)	Ubiquitous/Progenitor (*spi1*)	Embryonic	30%/50%	[78]
**MPD**	Dr *jak2a* ^*V581F*^	Ubiquitous	Embryonic	NS	[87]
	Dr *stat5* ^*H298R*/*N714F*^	Ubiquitous	Embryonic	NS	[88]
	Dr *etv6*-*jak2a* (*CML*)	Ubiquitous/Progenitor (*spi1*)	Embryonic	30%/50%	[78,89]
	Hs *kRAS* ^*G12D*^	Ubiquitous - inducible (hs + cre)	34 days (induced)	8%	[91]
	Hs *HRAS* ^*G12V*^	Endothelial (*fli1*)	Embryonic	NS	[92]
	Hs *NUP98*-*HOXA9*	Progenitor (*spi1*) - inducible (hs + cre)	19–23 months	23%	[97]
	Hs *KIT* ^*D816V*^	Ubiquitous	3–30 months	48–52 %	[93]
**MDS**	Dr *tet2*	Knockout	2 years	NS	[95]
	Dr *hspa9b*	Knockout	Embryonic	80%	[98]

Hs: *Homo sapiens*; Mm: *Mus musculus*; Dr: *Danio rerio*; AML: acute myeloid leukemia; ALL: acute lymphoblastic leukemia; MPD: myeloproliferative disease; MDS: myelodysplastic syndrome; NS: not specified; F0: founder; F1: first generation; F2: second generation; hs: heat shock; 4HT: 4-hydroxytamoxifen.

### Acute myeloid leukemia models

The first report using zebrafish as a model for leukemogenesis involved enforced expression of the human *RUNX1*-*ETO* oncogene, the product of the t(8;21)(q21;q22) translocation found in acute myelogenous leukemia (AML) [41]. Transient overexpression of this fusion gene in zebrafish embryos resulted in blood cell dysplasia, with increased numbers of blast cells, but reduced numbers of *c*-*myb*^+^ and *hbbe3*^+^ cells, along with circulatory defects [22]. This largely recapitulated the effects seen in mice [42], providing proof-of-principle evidence of the usefulness of this model. This study further showed that knockdown of zebrafish *runx1* produced a similar maturation arrest of blood progenitors that accumulated in the ICM, suggesting a likely dominant-negative effect of the RUNX1-ETO fusion protein on endogenous RUNX1 function, providing additional molecular insights [22]. Later, a stable transgenic line was generated that expressed *RUNX1*-*ETO* under the control of the zebrafish heat shock-inducible *hsp70* promoter. Induction of RUNX1-ETO elicited a fate change within the erythromyeloid lineage toward myelopoiesis, evident by upregulation of *spi1*^+^ and *mpo*^+^ cells at the expense of *gata1*^+^ cells. Overexpression of zebrafish *scl* rescued this phenotype, identifying suppression of SCL as an important mediator of RUNX1-ETO-induced reprogramming, as could treatment with the histone deacetylase inhibitor Trichostatin A [43]. These observations were consistent with both the transcriptional changes and therapeutic sensitivity reported in human AML patients carrying a RUNX1-ETO translocation [41], underscoring the value of zebrafish as a leukemic model. In subsequent chemical suppressor screens performed on this transgenic line, cyclooxygenase inhibitors [44] and the benzodiazepine Ro5-3335 [45] were identified as potential new therapeutics capable of antagonizing the leukemogenic function of RUNX1-ETO (Table 2).

**Table 2:** **List of chemical inhibitors screened in zebrafish models of leukemia**

**Chemical inhibitors**	**Mechanism**	**Gene**	**References**
**AML**			
Trichostatin A	Histone deacetylase inhibitor	*RUNX1*-*ETO*	[43]
Nimesulide	COX inhibitor	*RUNX1*-*ETO*	[44]
NS-398	COX inhibitor	*RUNX1*-*ETO*	[44]
Indomethacin	COX inhibitor	*RUNX1*-*ETO*	[44]
Benzodiazepine	RUNX1/CBFβ interactor	*RUNX1*-*ETO*	[45]
AC220	Tyrosine kinase inhibitor	*FLT3*-*ITD*	[49]
**T**-**ALL**			
Prephenazine	PP2A activator	*MYC*	[68]
LY294002	PI3K inhibitor	*pten* ^−/−^	[71]
AG490	JAK2 inhibitor	*etv6*-*jak2a*	[78]
**MPD**			
TG101209	JAK2 inhibitor	*jak2a* ^*V581F*^	[87]
AG490	JAK2 inhibitor	*etv6*-*jak2a*	[78]

COX: cyclooxygenase; CBFβ: core binding factor β; PP2A: protein phosphatase 2A; PI3K: phosphoinositide 3-kinase.

The first *bona fide* zebrafish model of AML was generated using human *MOZ*/*TIF2*, the oncogenic product of inv(8)(p11q13) observed in AML [46]. Transgenic expression of *MOZ*/*TIF2* under the control of the zebrafish white blood cell-specific *spi1* promoter led to the development of overt leukemia, characterized by an accumulation of immature myeloid cells in the kidney marrow, but decreased numbers of lymphocyte and precursor cells in the spleen. However, this occurred after a long latency period of 14 to 26 months, with a low incidence of ~1%, suggesting a role for additional genetic mutations [47].

Gain-of-function mutations in the receptor tyrosine kinase FLT3, including internal tandem duplications (FLT3-ITD) and amino acid substitutions in the tyrosine kinase domain (FLT3-TKD), are commonly found in AML [48]. Injection of mRNA encoding patient-derived FLT3-ITD or FLT3-TKD mutants in zebrafish led to constitutive activation of downstream signaling pathways, resulting in expansion of myeloid cells reminiscent of human AML. This was significantly ameliorated by the tyrosine kinase inhibitor AC220 in the case of FLT3-ITD, whereas the effects of FLT3-TKD were resistant to this agent [49].

Mutations in nucleophosmin (NPM1) that result in its aberrant relocation from nucleus to cytoplasm (termed NPMc^+^) represent one of the most common genetic alterations in adult AMLs [50]. Injection of mRNA encoding human NPMc^+^, but not NPM1, resulted in perturbation of primitive myelopoiesis, characterized by an increase in *spi1*^+^ myeloid precursors with later expansion of *mpo*^+^ and *csf1r*^+^ cells, but only in the absence of p53 [51]. This was consistent with the myeloproliferative phenotype reported in a transgenic mouse model expressing the human NPMc^+^ mutation [52]. Expression of NPMc^+^ in zebrafish also led to an increase in the number of both erythromyeloid precursors in the PBI and *c*-*myb*^+^/*cd41*^+^ HSCs in the ventral wall of the dorsal aorta, suggesting HSCs as the possible cellular origin for NPMc^+^ leukemia [51].

Members of the MYC family of transcription factors represent some of the most prevalent oncogenes in cancer, including myeloid malignancies, with MYCN overexpression frequently reported in AML patients with a poor prognosis [53]. Transgenic zebrafish expressing the murine *Mycn* gene under the control of the zebrafish *hsp70* promoter developed many aspects of human AML following induction of expression. This included enhanced cell proliferation and accumulation of immature blasts, increased myelopoiesis and anemia, with immature myeloblasts observed in the peripheral blood, kidney marrow, and spleen. Hematopoietic cell fate switch from the erythroid to the myeloid lineage in these transgenic fish was mediated by upregulation of *scl* and *lmo2*, leading to decreased expression of *gata1* and increased expression of *spi1* and *mpo*, along with *ndrg1*, a known MYCN target involved in granulocytic maturation [54].

Downregulation of *SPI1* expression has been consistently associated with AML [55,56]. Zebrafish carrying a hypomorphic *spi1* mutant allele exhibited overproduction of immature granulocytes in the CHT by 3 dpf and in the kidney marrow at later time points, which persisted to 18 months of age. This was associated with a reduction of lymphoid cells in kidney marrow and subsequent accumulation of myeloblasts in the peripheral blood, which closely mimicked aspects of human myelodysplastic syndrome (MDS)/AML. Interestingly, these mutant fish were sensitive to cytarabine, a chemotherapy agent used for the treatment of AML, which further validated the usefulness of zebrafish for therapeutic testing [57].

Finally, zebrafish have been used in a forward genetic screen to identify new genes involved in human hematologic malignancies. This identified a mutation in the *ddx18* gene that resulted in a loss of mature myeloid and erythroid cells due to increased apoptosis and p53-dependent G1 arrest. Screening of AML patients identified four non-synonymous sequence variants of DDX18. One of these showed a dominant negative effect over wild-type ddx18 when expressed in zebrafish, validating this approach [58].

### Acute lymphoblastic leukemia models

The first stable zebrafish leukemic model was generated by injection of DNA constructs in which sequences encoding mouse c-Myc were fused with those for enhanced green fluorescence protein (EGFP) and placed under the control of the zebrafish lymphoid-specific *rag2* promoter. This induced T cell acute lymphoblastic leukemia (T-ALL) with a latency of approximately 7 weeks, with an initial expansion of EGFP^+^ cells in the thymus, followed by dissemination of these cells into the kidney marrow, spleen, muscle, gut, gills, and fins. Gene expression analysis confirmed that this was the result of clonal expansion of transformed pre-T-lymphoblasts. When these cells were transplanted intraperitoneally into irradiated zebrafish, they homed back to the thymus before infiltrating other organs [2], further validating this as a *bona fide* leukemic model that has been subsequently utilized for a number of studies. For example, transplantation of the Myc-induced T-ALL cells into a syngenic zebrafish line without immune suppression identified a higher frequency of leukemia-initiating cells in T-ALL than previously thought, suggesting this may also be the case in human T-ALL [34]. This T-ALL syngenic zebrafish model was subsequently used for therapeutic testing, with both cyclophosphamine and vincristine showing efficacy in this model [59]. Collectively, these studies confirmed the similarities between zebrafish and mammalian T-ALL with respect to morphology, genetics and responsiveness to chemotherapic agents.

A conditional zebrafish transgenic line was generated from a similar expression construct, but with a loxed dsRED2 gene inserted upstream of the *c*-*Myc* oncogene. Induction of transgene expression by injection of mRNA encoding Cre-recombinase resulted in the development of T-ALL with a disease penetrance of ~13% [60]. Further work in this model demonstrated that enforced expression of zebrafish *bcl2* reduced the radiation sensitivity of these T-ALL cells [61]. Using a heat shock-inducible Cre-recombinase, disease penetrance was increased to 81% [62], with a follow-up study showing that co-expression of zebrafish *bcl2* in this model accelerated the initiation of T-lymphoblastic lymphoma (T-LBL) but inhibited progression to T-ALL [63], similar to mouse models [64,65]. These effects in zebrafish resulted from elevated levels of sphingosine-1-phosphate receptor 1 and intracellular adhesion molecule 1, which served to increase homotypic cell adhesion and block tumor cell intravasation [63].

An alternate T-ALL model used human MYC fused to a modified estrogen receptor, the nuclear transport of which was controlled by 4-hydroxytamoxifen. Induction of MYC translocation led to T-ALL, with withdrawal of 4-hydroxytamoxifen resulting in complete tumor regression in nearly 75% of fish. This model revealed that MYC suppressed the expression of the tumor suppressor PTEN, resulting in constitutive activation of the AKT pathway to promote tumor progression. Loss-of-function zebrafish *pten* mutations or expression of a constitutively-active Akt2 rendered tumors MYC-independent [66]. Further studies revealed these effects were mediated by the anti-apoptotic protein BIM [67]. Collectively, these studies both confirmed the importance of survival signals in ALL and suggested AKT pathway inhibitors as a new therapeutic strategy for T-ALL. A subsequent large-scale drug screen using this model identified prephenazine, an anticancer drug used to induce apoptosis in T-ALL cells, as an effective agent. Its effects were shown to be mediated by protein phosphatase 2A, suggesting that pharmacological activation of this enzyme may also have therapeutic potential [68].

The *PTEN* gene is also frequently mutated in hematological malignancies including T-ALL [69]. Zebrafish *pten*^−/−^ mutants showed leukemia-like phenotypes, including enhanced proliferation of HSCs within the CHT and differentiation arrest of committed progenitors across the erythroid, myeloid, and lymphoid lineages [70,71]. These phenotypes could be restored by pharmacological inhibition of PI3-K, known to be constitutively activated in the absence of PTEN, using LY294002 [71].

Activating mutations of NOTCH1 have been observed in nearly 60% of T-ALL patients [72]. Overexpression of a T-ALL-derived NOTCH1 intracellular domain mutant under the control of the zebrafish *rag2* promoter led to the development of a T cell lymphoproliferative disease. Neoplastic cells invaded tissues and caused a lethal leukemia when transplanted into irradiated recipients. Crossing of these fish with zebrafish overexpressing the *bcl2* gene dramatically accelerated the onset of leukemia, indicating a strong cooperation between the NOTCH1 and BCL2 pathways [73].

The *ETV6*-*RUNX1* oncogene, the product of t(1;19)(q23;p13), is frequently observed in B cell ALL [74]. Ubiquitous expression of human *ETV6*-*RUNX1* under the control of the zebrafish β-*actin* or Xenopus *ef*-*1* promoters resulted in expansion of lymphoid progenitors that resulted in oligoclonal B cell ALL at a frequency of ~3% after a latency period of 8–12 months. These fish showed a dramatic increase in total leukocyte count with more than 90% lymphoblasts observed in blood smears, with enlarged kidneys, the marrow of which were almost completely replaced with lymphoblasts, and dissemination of these cells into the brain, liver, muscle, and ovary, phenotypes reminiscent of childhood pre-B ALL in humans [74]. Lymphoblasts isolated from kidney marrow of these fish caused similar phenotypes in irradiated recipients between 6 and 9 weeks post transplantation, confirming the leukemic nature of these cells. This study identified pro-B precursors as the cellular target of ETV6-RUNX1 fusion, along with downregulation of the ETV6 tumor suppressor gene and deregulation of apoptotic genes, including an altered BCL2:BAX ratio, as potential molecular mediators of *ETV6*-*RUNX1*-induced leukemogenesis [75].

Other studies have shown that zebrafish gene orthologues can also recapitulate human oncogenes, further demonstrating conserved functionality across the two species. Alternative *ETV6*-*JAK2* fusions have been identified in both ALL and atypical chronic myelogenous leukemia (CML), that lead to constitutive activation of the JAK2 tyrosine kinase and downstream pathways [76,77]. A fusion was constructed from zebrafish *etv6* and *jak2a* genes corresponding to a human T-ALL-derived *ETV6*-*JAK2*. Transient expression of this *etv6*-*jak2a* fusion using either the zebrafish *spi1* promoter or the ubiquitous cytomegalovirus promoter caused specific disruption of lymphoid cells, with an increased number of *rag1*^+^ cells resulting in a fatal lymphoproliferative disorder [78]. The ETV6 gene has also been identified as a tumor-suppressor gene in lymphoid and other malignancies, with its function ablated by both epigenetic and genetic disruption, including the acquisition of truncating mutations [79]. Recent studies have shown that knockdown of zebrafish *etv6* resulted in a range of hematopoietic effects, including enhanced lymphopoiesis [80], while transient expression of truncated forms of the etv6 protein exerted similar effects, acting dominantly over the wild-type etv6 protein [81].

To identify other genetic lesions underlying T-ALL, a phenotype-driven forward genetic screen has been performed using chemical mutagenesis of transgenic zebrafish expressing EGFP^+^ in T lymphocytes. Several mutant lines were identified that recapitulated human T-ALL and T-LBL in both molecular and clinical features [82]. Iterative allo-transplantation of the T-ALL cells, selecting for aggressive disease that models relapsed human T-ALL, yielded leukemic clones that preserved the original genetic aberration in 55% of cases. However, these cells also acquired additional genetic aberrations that might be responsible for their enhanced malignant behavior. More importantly, over 50% of these genes were shared in relapsed human T-ALL samples, reiterating the similar molecular processes governing zebrafish and human hematopoietic malignancies [83].

### Myeloproliferative disorder models

Deregulation of the JAK/STAT signaling pathway is associated with a variety of myeloproliferative disorders (MPDs) [84,85]. For example, the somatic activating *JAK2*^*V617F*^ mutation is commonly observed in polycythemia vera and other myeloproliferative disorders [86]. Injection of mRNA encoding the equivalent zebrafish *jak2a*^*V581F*^ mutant resulted in enhanced erythropoiesis, with a significant increase in *gata1*^+^ precursors, concomitant with increased phosphorylation of the downstream stat5.1 transcription factor. Moreover, the effects of jak2a^V581F^ could be significantly ablated by application of a JAK2 inhibitor, paralleling the clinical situation, as well as knockdown of stat5.1, confirming the importance of this downstream pathway [87]. By corollary, transient expression of a constitutively-active zebrafish *stat5.1*^*H298R*^^/^^*N714F*^ mutant led to the expansion of early and late myeloid cells, erythrocytes, and lymphoid cells [88]. An *ETV6*-*JAK2* fusion gene has also been identified in atypical CML [76]. Expression of the corresponding zebrafish *etv6*-*jak2a* fusion gene using either the *spi1* or CMV promoter resulted in progenitor hyperproliferation, with increased numbers of immature myeloid and erythroid cells, perturbed mature myeloid cells and anemia, with the lymphoid lineage largely unaffected [89]. This contrasted with the phenotypes observed with the ALL-derived *etv6*-*jak2a* fusion, with the functional differences correlating with increased activation of the downstream STAT transcription factors by the atypical CML-derived *etv6*-*jak2a* fusion that also showed enhanced sensitivity to JAK2 inhibitors [78].

Activating mutations in *RAS* genes have been implicated in a number of human proliferative conditions, including the MPD juvenile myelomonocytic leukemia [90]. The first stable model of MPD-utilized heat shock-inducible Cre/Lox-mediated expression of human kRAS^G12D^ from the zebrafish β-*actin* promoter. Whole animal heat shock induction resulted in high tumor incidence with short latency, but associated with significant juvenile lethality and a propensity to develop other disorders, like rhabdomyosarcoma, intestinal hyperplasia, and malignant peripheral nerve sheath tumor. In contrast, *ex vivo* heat shock of kidney marrow cells specifically elicited a MPD when transplanted into sub-lethally irradiated recipient fish [91]. Expression of the HRAS^G12V^ oncogene from the zebrafish *fli1* promoter also recapitulated several pathologic aspects of MPD, including defective definitive hematopoiesis, with enhanced proliferation and infiltration of leukocytes into the CHT and kidney marrow. However, no perturbation of the lymphoid lineage was observed, highlighting the lineage-specific function of this oncogene. Expression of HRAS^G12V^ was also shown to downregulate NOTCH signaling, which contributed to the proliferation of erythromyeloid progenitors and disease pathogenesis [92].

Activating mutations in the receptor tyrosine kinase encoding *c*-*KIT* gene have been observed in systemic mastocytosis (SM), a rare MPD characterized by the infiltration of the clonally derived mast cells into a variety of different tissues including bone marrow [90]. Transgenic expression of the SM-derived *KIT*^*D816V*^ mutant under the control of the zebrafish β-*actin* promoter recapitulated features of aggressive SM, with infiltration of mast cells into the kidney marrow, associated with elevated expression of mast cell proteases, decreased *epcam* expression, and defects in cell-cycle progression, with significantly increased apoptosis [93].

### Myelodysplastic syndrome models

Mutation of the Ten-Eleven Translocation 2 (*TET2*) gene has been observed in MDS [94]. A *tet2*^−/−^ zebrafish line generated through genome editing demonstrated normal embryonic hematopoiesis, but developed a pre-myelodysplasia at 11 months of age. This culminated in true MDS at 24 months, characterized by increased progenitor and myelomonocytic cell numbers, and decreased erythrocyte cell numbers within the kidney marrow [95].

The *NUP98*-*HOXA9* oncogene, the product of t(7;11)(p15;p15), has been observed in MDS, CML blast crisis, and AML [96]. Conditional expression of the human *NUP98*-*HOXA9* oncogene under the control of the zebrafish *spi1* promoter was achieved using a Cre/lox-heat shock induction strategy. Heat shock-induced expression of *NUP98*-*HOXA9* perturbed hematopoiesis, with enhanced *spi1*^+^ myeloid precursors at the expense of *gata1*^+^ erythroid precursors. However, there was also disruption of myeloid differentiation, since mature *lcp*^+^, *lyz*^+^, and *mpo*^+^ myeloid cells were not expanded as much as the myeloid precursors. About 23% of the transgenic fish developed myeloproliferative neoplasms between 19 and 23 months, none of which progressed to AML [97].

Recently, the zebrafish crimsonless (*crs*) mutant was identified in a forward genetic screen that exhibited phenotypes consistent with MDS, including multilineage cytopenia, including decreased *hbbe3*^+^ and *mpo*^+^ cells, disrupted *gata1*^+^ precursors, and cell dysplasia. The causal mutation in *crs* was identified in *hspa9b*, encoding a conserved mitochondrial matrix chaperone, with the loss-of-function *hspa9b*^*G492E*^ mutation disrupting the substrate-binding domain of the protein. This resulted in perturbed mitochondrial function with increased production of reactive oxygen species (ROS) triggering oxidative stress and apoptosis of blood cells, leading to MDS [98].

## Conclusions

Zebrafish has emerged as a robust model for human leukemia and related disorders, including AML, ALL, MPD, and MDS. They have proven susceptible to a range of clinically relevant mutations, including those modeled on zebrafish genes, when expressed ubiquitously or in a lineage-specific manner. Alternatively, genetic screening has identified novel genes involved in these diseases. These models have provided unique insights into molecular and cellular aspects of disease etiology, as well as proving effective for therapeutic screening. However, there remains considerable scope for even greater use of this model. The transparency of this organism combined with exquisite transgenic and other fluorescent labeling approaches provides endless opportunities for high resolution *in vivo* imaging of tumor cell proliferation, dissemination, and interactions with the vasculature and other tissues [99]. The development of highly efficient genome editing approaches expands the potential to interrogate oncogenic and tumor-suppressor pathways and their interactions. In addition, a variety of xenotransplantation approaches are being developed [100], with the generation of immunodeficient and other useful zebrafish strains further enhancing the possibilities. These collectively provide increasingly advanced platforms for pre-clinical therapeutic development, including at the individual patient level, which will underpin advances in individualized medicine.

## Abbreviations

AGM: aorta-gonad-mesonephros
ALM: anterior lateral mesoderm
ALL: acute lymphoblastic leukemia
AML: acute myeloid leukemia
BP: B cell progenitor
CHT: caudal hematopoietic tissue
CLP: common lymphoid progenitor
CML: chronic myelogenous leukemia
CMP: common myeloid progenitor
EGFP: enhance green fluorescent protein
EMP: erythromyeloid progenitor
EP: erythroid progenitor
Ery: erythrocyte
G-CSFR: granulocyte colony-stimulating factor receptor
GMP: granulocyte-monocyte progenitor
Hemangio: hemangioblast
Hetero: heterophil
hpf: hours post fertilization
HSC: hematopoietic stem cell
ICM: intermediate cell mass
LBL: lymphoblastic lymphoma
MDS: myelodysplastic syndrome
Mono: monocyte/macrophage
MPD: myeloproliferative disorder
MPO: myeloperoxidase
NPM: nucleophosmin
PBI: posterior blood island
PLM: posterior lateral mesoderm
SM: systemic mastocytosis
TP: T cell progenitor
